# Supplementary material for: Causal Relationship between Aging and Anorexia Nervosa: A White-Matter-Microstructure-Mediated Mendelian Randomization Analysis
Source: Biomedicines. 2024 Aug 16;12(8):1874. doi: 10.3390/biomedicines12081874 (PMC11351342; doi:10.3390/biomedicines12081874)
Supplement: Supplementary file 1 [file biomedicines-12-01874-s001.zip › Supplementary Figure/Figure S7/Single SNP plot of MR analysis for ubm-b-1805 to AN.pdf]

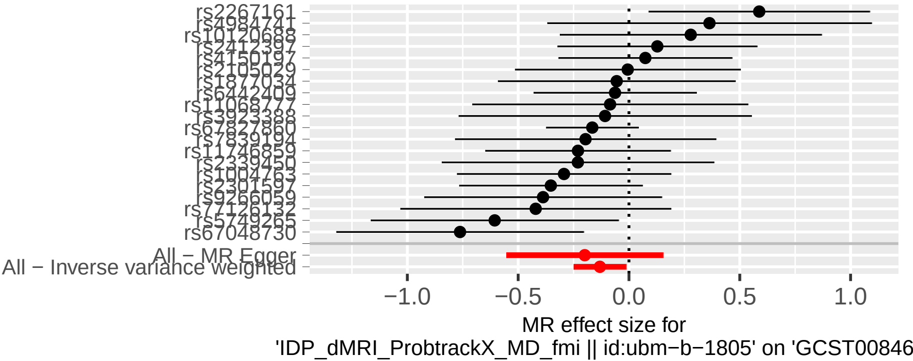
rs2267161
rs4984741
rs10120688
rs2412397
rs4150197
rs2105029
rs1877034
rs6442409
rs11068777
rs3923388
rs67827860
rs7839194
rs11746859
rs2339450
rs1004763
rs2301597
rs9266059
rs77126132
rs5749265
rs67048730
All − MR Egger
All − Inverse variance weighted
−1.0
−0.5
0.0
0.5
1.0
MR effect size for
'IDP_dMRI_ProbtrackX_MD_fmi || id:ubm−b−1805' on 'GCST00846
